# In Vivo Detection of *Staphylococcus aureus* Infections Using Radiolabeled Antibodies Specific for Bacterial Toxins

**DOI:** 10.1155/2024/3655327

**Published:** 2024-04-18

**Authors:** María Isabel González, Mario González-Arjona, Lorena Cussó, Miguel Ángel Morcillo, John Jairo Aguilera-Correa, Jaime Esteban, Martha Kestler, Daniel Calle, Carlos Cerón, Marta Cortes-Canteli, Patricia Muñoz, Emilio Bouza, Manuel Desco, Beatriz Salinas

**Affiliations:** ^1^Unidad de Medicina y Cirugía Experimental, Instituto de Investigación Sanitaria Gregorio Marañón (IiSGM), 28007 Madrid, Spain; ^2^Unidad de Imagen Avanzada, Centro Nacional de Investigaciones Cardiovasculares (CNIC), 28029 Madrid, Spain; ^3^CIBER de Salud Mental, Instituto de Salud Carlos III, 28029 Madrid, Spain; ^4^Unidad de Aplicaciones Médicas de las Radiaciones Ionizantes, Centro de Investigaciones Energéticas, Medioambientales y Tecnológicas (CIEMAT), 28040 Madrid, Spain; ^5^Departamento de Química en Ciencias Farmacéuticas, Universidad Complutense de Madrid, 28040 Madrid, Spain; ^6^Servicio de Microbiología Clínica Instituto de Investigación Sanitaria Fundación Jiménez Díaz, UAM, 28040 Madrid, Spain; ^7^CIBERINFEC-CIBER de Enfermedades Infecciosas, Madrid, Spain; ^8^Servicio de Microbiología y Enfermedades Infecciosas, Hospital General Universitario Gregorio Marañón, 28007 Madrid, Spain; ^9^Cardiovascular Risk Factors and Brain Function Programme, Centro Nacional de Investigaciones Cardiovasculares (CNIC) Carlos III, 28029 Madrid, Spain; ^10^Instituto de Investigación Sanitaria Fundación Jiménez Díaz (IIS-FJD), 28040 Madrid, Spain; ^11^Departamento de Medicina, Facultad de Medicina, Universidad Complutense de Madrid, 28040 Madrid, Spain; ^12^Departamento de Bioingeniería, Universidad Carlos III de Madrid, 28911 Madrid, Spain

## Abstract

**Purpose:**

The Gram-positive *Staphylococcus aureus* bacterium is one of the leading causes of infection in humans. The lack of specific noninvasive techniques for diagnosis of staphylococcal infection together with the severity of its associated complications support the need for new specific and selective diagnostic tools. This work presents the successful synthesis of an immunotracer that targets the *α*-toxin released by *S. aureus*.

**Methods:**

[^89^Zr]Zr-DFO-ToxAb was synthesized based on radiolabeling an anti-*α*-toxin antibody with zirconium-89. The physicochemical characterization of the immunotracer was performed by high-performance liquid chromatography (HPLC), radio-thin layer chromatography (radio-TLC), and electrophoretic analysis. Its diagnostic ability was evaluated in vivo by positron emission tomography/computed tomography (PET/CT) imaging in an animal model of local infection-inflammation (active *S. aureus* vs. heat-killed *S. aureus*) and infective osteoarthritis.

**Results:**

Chemical characterization of the tracer established the high radiochemical yield and purity of the tracer while maintaining antibody integrity. In vivo PET/CT image confirmed the ability of the tracer to detect active foci of *S. aureus*. Those results were supported by ex vivo biodistribution studies, autoradiography, and histology, which confirmed the ability of [^89^Zr]Zr-DFO-ToxAb to detect staphylococcal infectious foci, avoiding false-positives derived from inflammatory processes.

**Conclusions:**

We have developed an immuno-PET tracer capable of detecting *S. aureus* infections based on a radiolabeled antibody specific for the staphylococcal alpha toxins. The in vivo assessment of [^89^Zr]Zr-DFO-ToxAb confirmed its ability to selectively detect staphylococcal infectious foci, allowing us to discern between infectious and inflammatory processes.

## 1. Introduction

*Staphylococcus aureus* is one of the main causes of infectious morbidity and mortality worldwide. The bacterium is responsible for a significant percentage of cases of skin and soft tissue infection, pneumonia, bacteremia, infectious endocarditis (IE), and device-related infections [1, 2]. *S. aureus* is a major community and healthcare-associated pathogen, and its infections carry severe consequences for healthcare systems worldwide [3].

The severity of staphylococcal infections is, in part, a consequence of the ability of the bacterium to evade defensive barriers through the expression of virulent factors, such as toxins, enzymes, or surface proteins [4]. These elements are essential for the bacteria to colonize and invade the host tissue, enhance their adaptation to different environments in the host, and allow bacteria to avoid the immune system.

Toxins are exoproteins that represent key factors in bacterial aggressiveness [5, 6]. Among the different toxins released by *S. aureus*, alpha-toxin (*α*-toxin), also known as hemolysin (Hla), is secreted by 95% of clinical strains and constitutes the most virulent factor in infectious processes. There is scarce literature related to the pathogenic mechanism of *α*-toxin, although its fundamental role in staphylococcal infections has been widely demonstrated both in preclinical and clinical studies [7]. It is hypothesized that these toxins are abundantly released by *S. aureus* in the early stages of infection in order to establish an infectious niche and resist the action of the immune system, especially cells with a phagocytic mechanism [8]. These toxins are secreted in the form of soluble monomers able to bind specific membrane receptors on different cells or be absorbed into their lipid bilayer. In both cases, *α*-toxins form pores or channels in the cell membrane, which leads to cell lysis [9, 10]. Purportedly, once the infection is established, *α*-toxin secretion may decrease to prevent the generation of an increased immune response that could deteriorate the infectious niche [8].

After *S. aureus* colonizes and propagates through the tissues, e.g., soft tissues and bones, aided by virulent factors, the infection can lead to several complications. Its early identification is essential to determining the prognosis and optimizing clinical management. Current microbiological analyses employed in the definitive confirmation of infection and identification of the causative bacterial species involve collecting biological samples, such as blood, cerebrospinal fluid, or urine. However, the diagnosis of deep infectious foci requires much more invasive samples, such as aspiration or biopsies of the abscesses or infected bone, which is not always feasible in fragile critical patients. Noninvasive, rapid, and specific molecular imaging approaches for bacterial infections could address one clear unmet need in the diagnosis of infectious diseases.

Imaging has emerged as a noninvasive tool for the assessment of tissue damage. Structural imaging techniques, such as echography, X-ray, computed tomography (CT), or magnetic resonance imaging (MRI) are not capable of determining the type of microorganism causing the infection or whether it is alive. In the context of these limitations, nuclear imaging is highlighted as a promising tool due to its sensitivity and specificity for an accurate diagnosis of staphylococcal infections. Currently, 2-[^18^F]fluoro-2-deoxy-D-glucose ([^18^F]FDG) is one of the most sensitive imaging techniques for the noninvasive detection of infective foci in some important infectious-inflammatory pathologies, such as IE or osteomyelitis [11–13]. More than 50% of patients with endovascular infection by *S. aureus* (bacteremia or endocarditis) exhibit distant lesions when systematically scanned by [^18^F]FDG positron emission tomography (PET)/CT, which was the first technique to localize metastatic infectious foci and was successful in 30% of cases [14, 15]. This finding had a direct impact on the study of infections in distal lesions (in bone, spleen, or practically any organ) as this type of pathology involves prolonged treatment and sometimes requires surgical intervention to achieve complete eradication [16, 17]. Nevertheless, [^18^F]FDG is not able to efficiently differentiate between infectious foci, inflammatory processes, and tumoral lesions and may lead to confusion when bacterial foci are close to high-uptake organs, such as the heart or brain, limiting its potential use [18, 19]. So far, several studies have been published regarding the development of new imaging agents for the detection of infectious foci. Among other approaches, we can remark those based on small molecules and bacterial metabolites [20, 21], immuno-SPECT radiotracers [22], and those developed in the field of optical imaging [23]. However, limitations derived from a low target/nontarget ratio, burdensome preparation of precursors, or difficulties to clearly discern between infection and inflammation highlight the need to further address this problem.

In the development of novel approaches for more selective radiotracers, immuno-PET has emerged as a highly promising tool in diagnosis [24]. This technique based on the use of radiolabeled antibodies combines the high sensitivity of PET with the specificity of antibodies towards the targeted cell, tissue, or receptor. Although this noninvasive technology has achieved satisfactory results in oncology [25, 26], its application to infectious diseases is still considered promising but largely unexplored, as only a few radioimmune tracers have been developed in recent years. While some of these antibody-based probes react indistinctly against surface receptors from Gram-positive bacteria [27], others are able to specifically target antigens superficially presented by *S. aureus* [23].

In the present study, we present the synthesis and in vivo evaluation of an immuno-PET tracer based on zirconium-89 (^89^Zr) radiolabeling of a commercial anti-alpha toxin antibody for the noninvasive, selective, and specific in vivo detection of staphylococcal infections by nuclear imaging.

## 2. Materials and Methods

Unless otherwise noted, all reagents were purchased from Merck Life Science (Darmstadt, Germany). Rabbit anti-Hla pAb *S. aureus* alpha-toxin polyclonal antibody (*α*-ToxAb) was acquired from IBT BioServices (Rockville, MD, USA). The isothiocyanate-bearing derivative of desferrioxamine (p-DFO-Bz-NCS) was purchased from CheMatech (Dijon, France). [^89^Zr]Zr-oxalic acid solution and [^18^F]FDG were purchased from PerkinElmer (Waltham, MA, USA) and Curium Pharma (Madrid, Spain), respectively.

### 2.1. Synthesis of Radiolabeled Anti-Alpha-Toxin Antibody

The ^89^Zr-ToxAb immunotracer was synthesized by adapting protocols published elsewhere [28, 29].

#### 2.1.1. Synthesis of Bz-DFO-ToxAb

As a first step, we conjugated the antibody to the chelator desferrioxamine (p-DFO-Bz-NCS). We concentrated 1 mg/mL of *α*-ToxAb using 10 KDa Amicon filters, and the recovered product was adjusted to 1 mL with 0.1 M sodium bicarbonate buffer (pH 9.0). Separately, p-DFO-Bz-NCS was dissolved in dimethyl sulfoxide and 20 *μ*L (2% *v*/*v*) was added to the antibody solution, corresponding to a 5-fold molar excess of chelator. The mixture was allowed to react for 30 min at 37°C. The reaction mixture was purified using a PD-10 desalting column (GE Healthcare Bio-Science AB, Chicago, IL, USA). The isolated product, DFO-ToxAb, was collected in 2 mL of 0.5 M HEPES and stored at -20°C until radiolabeling.

#### 2.1.2. Radiolabeling of [^89^Zr]Zr-DFO-ToxAb

Based on previous reports [28], a [^89^Zr]Zr-oxalic acid solution (74–111 MBq) was filled up to 200 *μ*L by adding 1 M oxalic acid. Next, 90 *μ*L of 2 M sodium carbonate was added, and the mixture was incubated for 3 min at room temperature. Consecutively, 0.30 mL of 0.5 M HEPES, 0.71 mL of Bz-DFO-ToxAb, and 0.70 mL of 0.5 M HEPES were pipetted into the reaction tube, and the mixture was incubated for 1 h at room temperature. The reaction product was purified by centrifuging with 10 KDa Amicon filters, and the radiochemical yield was estimated as the ratio between radioactivity measured on recovered [^89^Zr]Zr-DFO-ToxAb and the initial amount of nuclear activity.

The specific activity was expressed as the radioactivity measured on pure [^89^Zr]Zr-DFO-ToxAb versus the final mass of the antibody (MBq/mg). The antibody concentration was determined by Bradford-Coomassie assay according to the manufacturer's instructions and employing a Synergy™ HT Multi-Mode Microplate Reader (Biotek Instruments Inc., Winooski, VT, USA).

### 2.2. MALDI-TOF MS/MS Analysis

To determine the molar ratio of p-DFO-Bz-NCS to *α*-ToxAb, commercial antibody and Bz-DFO-ToxAb were analyzed by MALDI-TOF MS/MS (Unidad de Espectrometría de Masas, Universidad Complutense de Madrid, Spain) according to protocols published elsewhere [30, 31]. Briefly, 1 *μ*L of each antibody sample was mixed with 1 *μ*L of sinapic acid as matrix solution (10 mg/mL in 50% acetonitrile: water and 0.1% trifluoroacetic acid). The mixture was placed over a stainless-steel target plate and allowed to air dry. After determining the mass (m/z) of the unmodified antibody and the immunoconjugate, the difference was divided by the molecular weight of the chelator.

### 2.3. High-Performance Liquid Chromatography (HPLC) and Radio-Thin Layer Chromatography (Radio-TLC)

An Agilent 1200 series HPLC system equipped with a Gina Scan-RAM Radio-TLC/HPLC detector (Microbeam S.A., Madrid, Spain) was employed for determining the radiochemical purity (percent of total radioactivity which is present as the desired radiolabeled species) of the [^89^Zr]Zr-DFO-ToxAb. Analytic runs were performed with a Yarra SEC-3000 column (Phenomenex Inc., Torrance, CA, USA) under an isocratic gradient of phosphate-buffered saline (1x PBS) between 0 and 80 min, with a flow of 0.2 mL/min. Quality control was performed by radio-TLC using a miniGita Single system (Elisa-Raytest, Angleur, Belgium), Whatman™ strips (3 MM CHR; GE Healthcare Bio-Science AB, Chicago, IL, USA) as the stationary phase, and citric acid monohydrate/sodium carbonate (pH 4.9-5.1) as the eluent.

The radiochemical stability of the immunotracer was assessed by incubation in 1x PBS at 37°C. Aliquots (*n* = 3) were collected at different time points between 0 and 72 h (1, 2, 4, 19, 20, 22, 24, 28, 44, 48, 51, 68, and 72 h) and analyzed by radio-TLC under the experimental conditions discussed above.

### 2.4. Sodium Dodecyl Sulfate-Polyacrylamide Gel Electrophoresis (SDS-PAGE)

Briefly, 5 *μ*g of each antibody sample (commercial *α*-ToxAb, Bz-DFO-ToxAb, and [^89^Zr]Zr-DFO-ToxAb) was mixed with loading buffer under reducing conditions to preserve antibody integrity. Mixtures were boiled for 6.5 min at 95°C and then loaded onto a 7.5% sodium dodecyl sulfate-polyacrylamide gel along with standard molecular weight markers (Precision Plus Protein Dual Xtra Standards; Bio-Rad, Hercules, CA, USA). Samples were run at 90 V for 2.5 h before staining the gel with Coomassie Blue (Bio-Rad, Hercules, CA, USA) for 45 min. The gel was destained with a solution of 50% methanol, 40% acetic acid, and 10% water for 24 h.

### 2.5. In Vivo Pharmacokinetic Evaluation

We administered 9.25–11.10 MBq of [^89^Zr]Zr-DFO-ToxAb (300 *μ*L) via intravenous (i.v.) injection to three wild-type Sprague-Dawley (SD) rats. Radioactivity was measured in serial blood samples extracted from the saphenous vein of awake rats at different time points postinjection (10, 20, 30, 50, 60, 75, 90, 105, 120, 270, 300, 330, 400, 1440, 1560, 1850, 2790, 3050, 4200, and 4320 min). Radioactivity per sample was measured using a Wallac Wizard 1480-011 Automatic Gamma Counter (PerkinElmer, Waltham, MA, USA), and measurements were presented as the mean of percent injected dose per milliliter of sample (%ID/mL).

Pharmacokinetic parameters were obtained from the %ID/mL using a two-compartmental analysis model and the PKSolver add-in program for Microsoft Excel [32, 33]. To apply this analysis, experimental values were selected or discarded following the Akaike information criterion (AIC), a number score calculated during data processing to improve the fit of these values to the selected kinetic model.

### 2.6. Bacterial Culture

To prepare the bacterial dose, a *S. aureus* strain from the American Type Culture Collection (ATCC 29213) was seeded and grown in tryptic soy broth (TSB) for 24 h before in vivo inoculation. Bacteriological suspensions were then prepared at a concentration of 10^8^ CFUs/mL, adjusting the McFarland turbidity standards at 0.5.

### 2.7. Ethical Statement for Animal Experiments

All applicable international, national, and/or institutional guidelines for the care and use of animals were followed. All animal procedures complied with the ARRIVE guidelines and conformed to EU Directive 2010/63EU and national regulations (RD 53/2013). All animal procedures were approved by the HGUGM Animal Experimentation Ethics Committee and by the Animal Protection Board of the Comunidad Autónoma de Madrid (PROEX 083/18). Animals were housed in the animal facility of Hospital General Universitario Gregorio Marañón (HGUGM), Madrid, Spain (ES280790000087).

### 2.8. In Vivo Assessment of [^89^Zr]Zr-DFO-ToxAb in Staphylococcal Infection-Inflammation Rat Model

#### 2.8.1. Infection-Inflammation Rat Model

In vivo assessment of the novel radiotracer was performed in a soft tissue bacterial infection model employing female Sprague-Dawley (SD) rats (6-8 weeks old; 250-300 g). The infection-inflammation animal model was induced following protocols published elsewhere [34]. SD rats were intramuscularly injected in the right thigh with live *S. aureus* (ATCC SAMS: 5 × 10^7^ CFU) and in the left thigh with heat-killed *S. aureus* (ATCC SAMS: 5 × 10^7^ CFU), representing infectious and inflammatory tissues, respectively. Animals were kept anesthetized under 1.5% sevofluorane in oxygen (SevoFlo, Zoetis Belgium SA, Louvain-la-Neuve, Belgium), and analgesia and postoperative care were not required.

Animals were divided into groups as follows: 9 underwent PET imaging of ^89^Zr-ToxAb, 6 in biodistribution studies, 3 in autoradiography assays, 4 underwent [^18^F]FDG-PET imaging, 3 underwent free [^89^Zr]Zr-oxalate imaging, and 5 underwent microbiological assessment and histology.

#### 2.8.2. PET/CT Imaging of [^89^Zr]Zr-DFO-ToxAb in the Staphylococcal Infection-Inflammation Rat Model

The ability of immunoconjugates to detect active staphylococcal foci was evaluated by PET/CT imaging in the staphylococcal infection-inflammation rat model (*n* = 9). PET/CT scans were acquired 24 h after inoculation using a small-animal PET/CT scanner (PET/CT SuperArgus, SEDECAL, Madrid, Spain). Longitudinal scans were obtained at 1, 24, and 48 after i.v. injection of [^89^Zr]Zr-DFO-ToxAb (9.25–11.10 MBq, 300 *μ*L, PBS). During the acquisition, animals were kept anesthetized under 1.5% sevofluorane in oxygen (SevoFlo, Zoetis Belgium SA, Louvain-la-Neuve, Belgium). PET data were collected for 40 min in two-bed positions (20 min per bed) and reconstructed using OSEM-2D with 16 subsets and 1 iteration (voxel size: 0.388 × 0.388 × 0.775 mm). After the PET scan, a CT scan was performed with an X-ray beam current of 340 *μ*A and a tube voltage of 40 kVp. Scans were reconstructed using an FDK algorithm [35].

Regions of interest (ROIs) were manually drawn on the CT over the hind legs and then applied to the coregistered PET images. ROIs delimited the area of soft tissue, excluding bone and bone marrow. The same criteria were applied to both legs. The total activity measured was corrected by the injected dose, animal weight, and isotope decay. Quantitative results were expressed in absolute form as the Log10 (activity) or as a ratio (infected-to-inflamed tissue) of these log-transformed values. This procedure ensured data normality, due to the geometric growth of bacteria.

#### 2.8.3. [^18^F]FDG PET/CT Imaging in the Staphylococcal Infection-Inflammation Rat Model

In vivo [^18^F]FDG PET-CT scans were acquired employing the soft tissue staphylococcal infection-inflammation model (*n* = 4) 72 h after bacteria inoculation. PET/CT images were acquired 2 h after the injection of 37–48 MBq [^18^F]FDG (300 *μ*L). We used the acquisition and imaging quantification protocols described above for the imaging of the infection-inflammation model.

#### 2.8.4. [^89^Zr]Zr-Oxalic Imaging in the Staphylococcal Infection-Inflammation Rat Model

In vivo imaging of [^89^Zr]Zr-oxalic in the staphylococcal infection-inflammation rat model was performed as the control group, 24 h and 48 h after free radionuclide administration (*n* = 3), by the i.v. injection of 7.25–8.00 MBq (300 *μ*L PBS), and also following the same acquisition protocol described above for the imaging of the infection-inflammation model.

#### 2.8.5. Ex Vivo Biodistribution Studies of [^89^Zr]Zr-DFO-ToxAb

Parallel to the in vivo imaging studies, the same staphylococcal infection-inflammation rat model was used for ex vivo assessment of the biodistribution of [^89^Zr]Zr-DFO-ToxAb 1, 24, and 48 h after i.v. tracer injection via the lateral tail vein (300 *μ*L, 10 MBq). Six rats were euthanized at each time point, and the organs of interest (brain, heart, lungs, liver spleen, kidneys, inoculated soft tissues, and blood) were harvested. The activity per collected organ was measured using a Wallac Wizard 1480-011 Automatic Gamma Counter and expressed as the mean %ID/g.

#### 2.8.6. Ex Vivo Autoradiography of [^89^Zr]Zr-DFO-ToxAb in Muscles

After in vivo imaging, three staphylococcal infection-inflammation rats were sacrificed and the hind leg muscles were excised. Immediately after tissue collection, the fresh samples were carefully sectioned using a manual device consisting of a small tissue fixation chamber and blades. Series of 100 - 200 *μ*m sections were sliced, mounted on plastic slides, and placed on a digital autoradiography phosphor plate (BAS-MP 2025, Fujifilm, Tokyo, Japan) for 30 min. The imaging plates were read at a pixel resolution of 200 *μ*m with a Bio-Imaging Analyzer BAS-500 plate reader (Fujifilm, Tokyo, Japan). To quantify tissue uptake, we used Fiji ImageJ software (U.S. National Institutes of Health, Bethesda, MD, USA).

#### 2.8.7. Ex Vivo Evaluation of Infected and Inflamed Soft Tissues by Microbiological Culture and Histology

To confirm staphylococcal infection, five rats from the soft tissue staphylococcal infection-inflammation model were euthanized 72 h after bacteria inoculation, and the targeted soft tissues were harvested under aseptic conditions and routinely processed by mincing and grinding using a sterile mortar with saline. Next, 100 *μ*L of the samples was qualitatively extended over a blood agar plate and cultured for 24 h at 37°C. Bacterial colonies were identified by mass spectrometry employing a Bruker Daltonik MALDI Biotype system (Bruker, Billerica, MA, USA).

Additionally, immunofluorescent staining of control noninfected and *S. aureus*-infected soft tissues was performed to confirm the microbiological results. Briefly, harvested muscles were fixed with 4% PFA, dehydrated with sucrose, and embedded in optimal cutting temperature compound (OCT). After cutting in layers, mounted slides were incubated with *Staphylococcus aureus* primary polyclonal antibody (1 : 100, #PA1-7246, Fisher Scientific, Waltham, MA, USA) and goat anti-rabbit IgG (*H* + *L*) secondary antibody (1 : 400, Alexa Fluor™ 488, #A-11008, Fisher Scientific, Waltham, MA, USA). Then, DAPI dye (1 : 2000) was applied over the samples, and coverslips were placed over the slides using a DAKO mounting medium (Agilent Technologies, Santa Clara, CA, USA). Fluorescent-stained tissues were scanned using an Axio Scan Z1, and images were analyzed with the ZEN Blue software (both from Zeiss, Oberkochen, Germany).

#### 2.8.8. Immunohistochemistry of Macrophages

Three animals from the soft tissue staphylococcal infection-inflammation model were euthanized 24 h after inoculation, as well as 1 wild-type rat used as a control. Soft tissue from the left and right hind legs was harvested, fixed in 10% formalin, and embedded in paraffin. Tissues were serially sectioned at 4 *μ*m and deparaffinized. Antigen retrieval was performed on tissue sections using PTLink at pH 6, followed by peroxidase blocking and further blocking with 1% bovine serum albumin (BSA) and 1% normal goat serum (Sigma-Aldrich, St. Louis, MO, USA). After incubation with primary antibody (rat anti-F4/80, 1 : 1500, ab6640; Abcam, Cambridge, UK), samples were treated with secondary antibody (rabbit anti-rat, P0450) and Envision HRP. 3,3′-Diaminobenzidine (DAB) chromogen was then applied, and the tissues were counterstained with hematoxylin. The presence of inflammatory cells due to live or heat-killed *S. aureus* inoculation was also confirmed by F4/80 immunohistochemistry.

Images were digitized using an Axio Scan Z1 and were analyzed with the ZEN Blue software (both from Zeiss, Oberkochen, Germany).

### 2.9. Validation of [^89^Zr]Zr-DFO-ToxAb in an Infective Osteoarthritis Mouse Model

#### 2.9.1. Infective Osteoarthritis Mouse Model

An infective osteoarthritis model employs female RjOrl:SWISS (CD1) mice (*n* = 4, 30-32 g, 11 weeks old). This model was induced following the methodology described by Aguilera-Correa et al. [36]. Briefly, female RjOrl:SWISS (CD1) mice (*n* = 4, 30-32 g, 11 weeks old) underwent surgery which consisted of inserting a 1 cm long resorbable implant (Biosyn 0; Synature) into the medullary cavity of the right femur through the joint. The implant was previously infected with 10^8^ CFUs/mL inoculum of *S. aureus*. The left knee of mice was considered a healthy control model. After surgery, animals were given ibuprofen in drinking water (0.5 mg/mL) and hamster food (Vital Menu, Vitakraft, Germany) for the duration of the study. Mice were also monitored for clinical conditions (lameness, piloerection, lack of grooming, wounds, passivity, and aggressiveness) until the end of the study.

#### 2.9.2. In Vivo PET/CT Imaging of [^89^Zr]Zr-DFO-ToxAb in the Infective Osteoarthritis Mouse Model

The longitudinal study of the prognosis of osteoarthritis using our immunotracer was carried out over 6 weeks, with tracer injections every 14 days. CD1 mice (*n* = 4, 30-32 g, 11 weeks old) were intravenously administered with [^89^Zr]Zr-DFO-ToxAb (4.18-5.03 MBq, 200 *μ*L, PBS) every 14 days through the lateral tail vein, and static scans were recorded at 1 week, 2 weeks, 3 weeks, 4 weeks, 5 weeks, and 6 weeks postsurgery. As [^89^Zr]Zr-DFO-ToxAb was administered every 14 days, the radiotracer uptake was 48 h in the images at 1, 3, and 5 weeks and 7 days in the images at 2, 4, and 6 weeks (Figure S5). Animals were placed in a prone position, and the field of view was adjusted to the area of interest. PET data were acquired for 30 min and reconstructed using OSEM-2D with 16 subsets and 1 iteration (voxel size: 0.388 × 0.388 × 0.775 mm). For the anatomical image acquisition, CT parameters selected were 40 KeV, 340 *μ*A, 360 projections and binning 2 × 2, and 0.12 mm^3^ voxel size. The images were reconstructed in the same way as described above for the staphylococcal infection-inflammation rat model.

#### 2.9.3. Histological and Microbiological Validation of the Infective Osteoarthritis Mouse Model

Animals were sacrificed after the last imaging time point, and the infected femur of the mice was removed. Half of the samples were intended for histological studies and the other half for microbiological analysis. Briefly, histological preparation consisted of fixation of samples in 10% buffered formaldehyde for 24 h, and subsequent decalcification in the Surgipath Decalcifier (Leica, Wetzlar, Germany) for 12 h. After confirming the correct decalcification of the material, it was carved, and the joint and surrounding soft tissues were embedded in paraffin. The paraffin-embedded material was sectioned at 3 microns, stained with hematoxylin and eosin (H&E) for histological assessment, and with Gram's stain to assess the presence of germs. The degree of inflammation of the muscle, periarticular soft tissue, bone, joint, and bone/joint was assessed on a scale of 0 to 3 for the samples. In addition, the number of Gram+ germs was assessed qualitatively. The assessing pathologist was blinded to the type of intervention performed. For microbiological analysis, each dissected femur was crushed with a hammer inside a sterile plastic bag. The resulting crushed tissue was sonicated in 2 mL of 0.9% NaCl (B. Braun) and sonicated in a JP Selecta sonicator for 5 minutes at room temperature. The resultant liquid was seeded using the spread plate technique onto chocolate-blood agar (bioMérieux, Îlle de France, France). The resulting sonicated samples were diluted in saline to a concentration of 1 : 10, 1 : 100, and 1 : 1,000 before being seeded onto an agar plate in the amount of 100 *μ*L and spreading with a Digralsky loop until fully absorbed. For 48 hours, all plates were incubated at 37°C with 5% CO_2_. By counting viable colonies, the final bacterial concentration was calculated and expressed in CFU per gram of bone and adnexa.

### 2.10. Data Processing and Statistical Analysis

Data were processed and graphs were created using Prism 8.3.0 (GraphPad Software, La Jolla, CA, USA).

Repeated measures analysis of variance (ANOVA) was used to assess the progression of infection vs. inflammation with a simple contrast using the 1-hour value as a reference. The paired Student *t*-test was used to compare the infection : inflammation ratio at each time point. These data satisfied the assumptions of normality and homogeneity of variance. The Mann–Whitney *U* test was used to compare [^18^F]FDG vs. [^89^Zr]Zr-DFO-ToxAb (which did not comply with those assumptions). All data are reported as mean (± standard deviation), and the threshold for significance was set at *p* < 0.05.

To evaluate the ex vivo biodistribution data, repeated measures ANOVA and Tukey's multiple comparison test were applied after checking for normality. The significance threshold was also set to *p* < 0.05.

## 3. Results

### 3.1. Characterization of [^89^Zr]Zr-DFO-ToxAb

**Figure 1:**
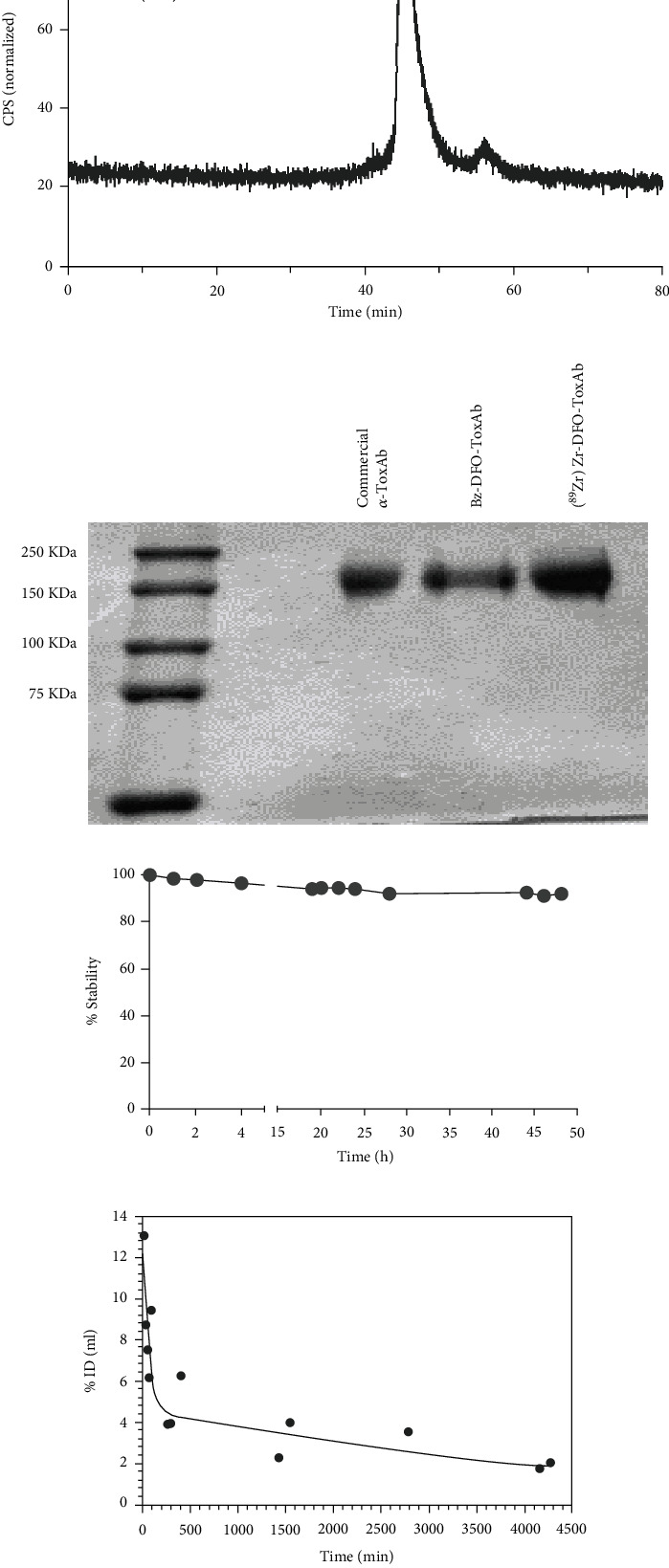
Characterization of [^89^Zr]Zr-DFO-ToxAb. (a) High-performance liquid chromatography (HPLC) and (b) electrophoretic analysis. (c) Radiochemical stability (*n* = 3) was assessed by radio-thin layer chromatography (radio-TLC). (d) In vivo circulation time in the blood (*n* = 3) measured by the gamma counter.

Employing standard published protocols for the synthesis of radiolabeled antibodies [28, 29], [^89^Zr]Zr-DFO-ToxAb radiotracer was synthesized with a radiochemical yield of 78.55 ± 1.79%, a specific activity of 534.68 MBq/mg, and a radiochemical purity of 96.45% (Figure 1(a) and Figure S1). SDS-PAGE confirmed the integrity of the antibody in the different preparations, maintaining its original molecular weight and discarding its fragmentation or dimerization (Figure 1(b)).

Mass spectrometry revealed that 1.12 ± 0.05 Bz-DFO moieties were coupled per antibody molecule. In vitro stability studies in PBS showed values of 90.37% after 2 days (Figure 1(c)).

### 3.2. Pharmacokinetics and Ex Vivo Biodistribution of [^89^Zr]Zr-DFO-ToxAb

The terminal phase half-life of the [^89^Zr]Zr-DFO-ToxAb determined by a two-compartment distribution model was 2.33 days (Figure 1(d)), matching the circulation time of IgGs [37]. This model also allowed calculation of the volume of distribution in the steady state and systematic clearance of the tracer, which were 20.37 mL (76.87 mL/kg) and 6.22 mL/d (23.47 mL/d/kg), respectively.

**Figure 2:**
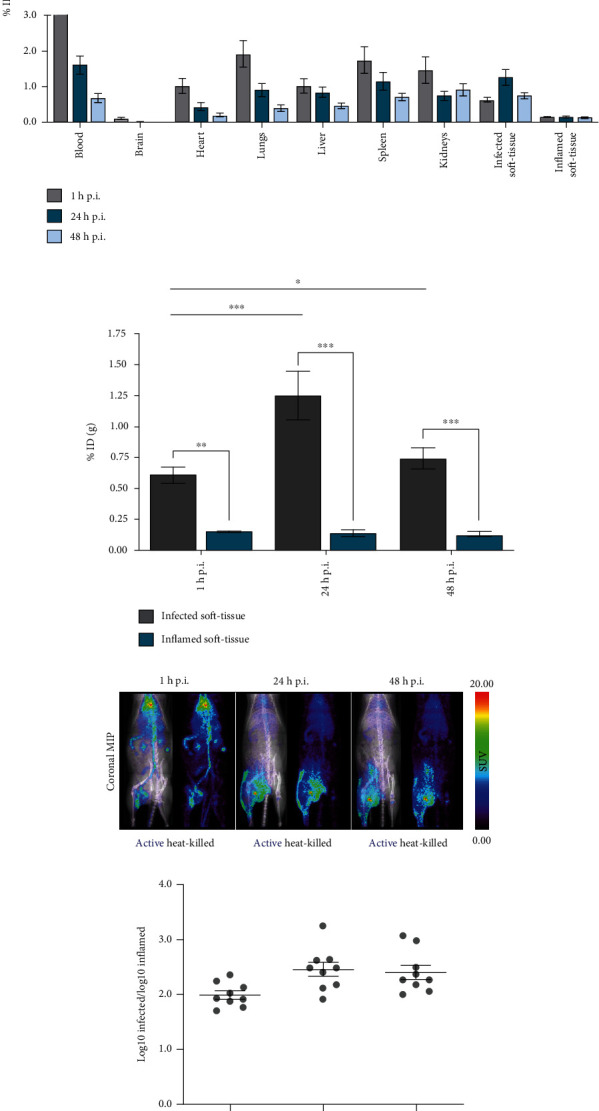
In vivo evaluation of [^89^Zr]Zr-DFO-ToxAb and ex vivo biodistribution. (a) The ex vivo biodistribution in organs of interest (*n* = 6) was measured at 1, 24, and 48 h after injection of the radiotracer and expressed as the mean of percent injected dose per gram of tissue (%ID/g). (b) Statistical analysis of the ex vivo biodistribution in infected (blue) and inflamed (grey) soft tissues (*n* = 6; ANOVA, *p* < 0.05). (c) Whole body coronal maximum intensity projections (MIPs) (*n* = 9). (d) Region of interest quantification is presented as the ratio of Log10-infected to Log10-inflamed over time (*n* = 9). Data are expressed as the mean ± standard deviation, and the threshold for significance was set at ^∗^*p* < 0.05, ^∗∗^*p* < 0.01, and ^∗∗∗^*p* < 0.001.

Ex vivo biodistribution analysis (*n* = 6) revealed renal and hepatobiliary clearance, with the main uptake in the liver (1.03 ± 0.45%ID/g), spleen (1.73 ± 0.94%ID/g), and kidneys (1.45 ± 0.94%ID/g) in the short-term (1 h postinjection; Figure 2(a)). A progressive decrease in activity was observed over time in these organs (0.47 ± 0.15%ID/g in liver, 0.70 ± 0.19%ID/g in spleen, and 0.91 ± 0.42%ID/g in kidneys, 48 h postinjection).

Ex vivo biodistribution (Figure 2(b)) also demonstrated a higher accumulation of [^89^Zr]Zr-DFO-ToxAb in the infected muscle compared to the inflamed contralateral tissue (Table S1), with significant differences at all time points and maximal values of 1.25 ± 0.83%ID/g in the infected region at 24 h (repeated measures ANOVA, *p* < 0.05).

### 3.3. In Vivo PET/CT Assessment of [^89^Zr]Zr-DFO-ToxAb in Staphylococcal Infection-Inflammation Rat Model

PET imaging (*n* = 9) revealed high uptake of the radiotracer [^89^Zr]Zr-DFO-ToxAb in the infection region, even at early time points (1 h postinjection), increasing up to 24 h, after which it remained stable (repeated measures ANOVA, *p* < 0.05; Figure 1(c) and Figure S2). ROI quantification confirmed a higher accumulation of the radiotracer in the infected muscle compared to the contralateral area inoculated with the heat-killed bacteria, reaching a ratio of Log10-infected to Log10-inflamed of 1.99 ± 0.27 at 1 h, which increased up to 2.41 ± 0.38 at 48 h (paired Student *t*-test, *p* < 0.05; Figure 2(d)). Whole-body PET-CT scans confirmed the localization of [^89^Zr]Zr-DFO-ToxAb uptake in the infection focus, which was noticeably higher than in other organs, including the liver, spleen, and lungs (Figure 2(c)).

### 3.4. Ex Vivo Autoradiography of [^89^Zr]Zr-DFO-ToxAb in Staphylococcal Infection-Inflammation Rat Model

**Figure 3:**
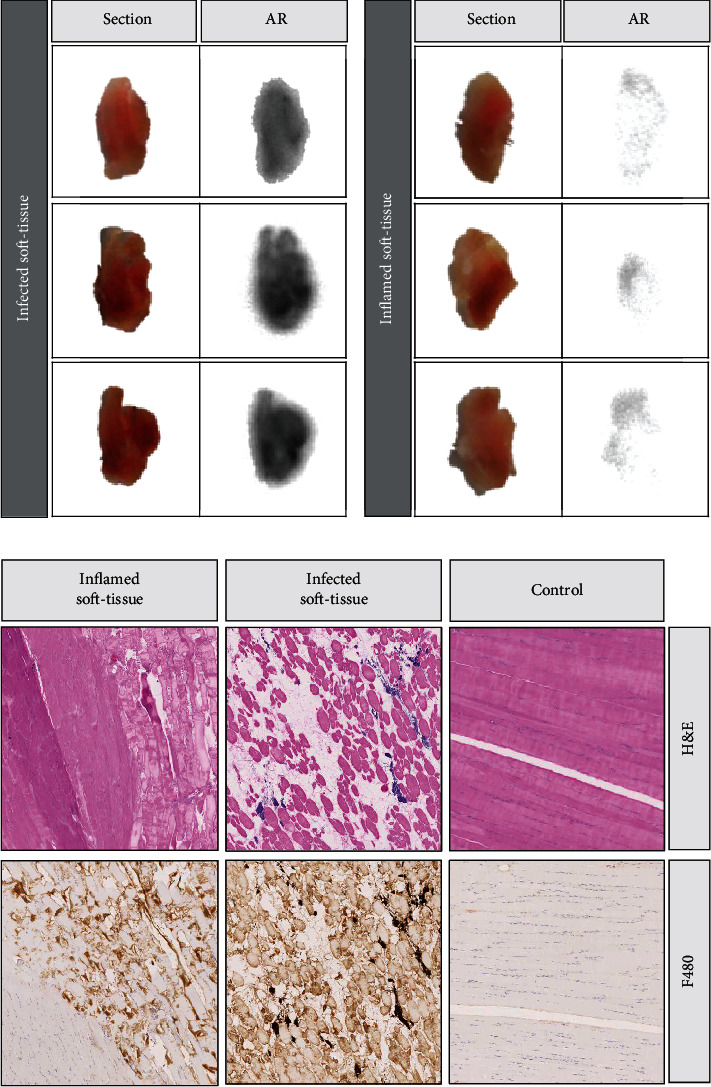
Ex vivo analysis of infected and inflamed tissues. (a) Tissue tracer uptake was analyzed by ex vivo autoradiography (*n* = 3). (b) The presence of macrophages (brown color) and soft tissue damage was assessed by hematoxylin and eosin (H&E) staining and F4/80 immunohistochemical analysis (*n* = 3).

Autoradiography of infected and inflamed tissue revealed a 3-fold higher radiotracer uptake in the infected tissue compared to the inflamed tissue, together with low uptake of the macrophages in the inflamed region (Figure 3(a)).

### 3.5. Ex Vivo Evaluation of Infected and Inflamed Soft Tissues by Microbiological Culture and Histology

The microbiological culture of the homogenized tissues confirmed the presence of *S. aureus* in the excised tissues. Effective induction of infection in the left hind legs of the animals was also checked by confocal imaging, which showed the presence of *S. aureus* in the stained tissues (Figure S3).

Immunohistochemical analysis confirmed the existence of inflammation in both legs of the rats (Figure 3(b)). In the leg infected with alive *S. aureus*, H&E staining showed ruptured muscle fibers as well as an accumulation of immune and inflammatory cells, such as neutrophils or macrophages, around the damaged muscle structures. Although this damage was lower in the noninfectious inflamed soft tissue (administered with heat-killed *S. aureus*), tissue sections also showed the presence of inflammatory cells both between the muscle fibers and especially in the fat clusters of the soft tissue.

Although F4/80 staining stains nonspecifically for collagen structures within the muscle tissue, the staining of macrophages (brown color) in the inflamed and especially in the infected soft tissue confirmed the presence of these cells in both cases (Figure 3(b)).

### 3.6. In Vivo PET/CT Imaging of [^18^F]FDG in Staphylococcal Infection-Inflammation Rat Model

**Figure 4:**
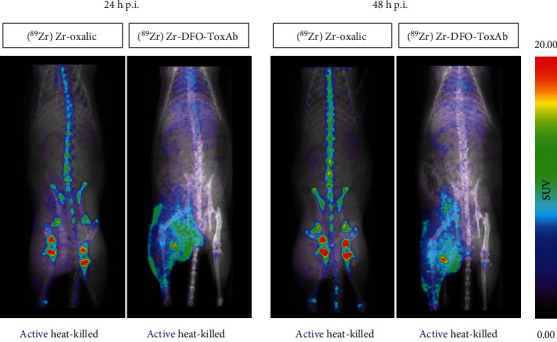
Comparison of [^89^Zr]Zr-oxalic and [^89^Zr]Zr-DFO-ToxAb tracers in local infection-inflammation model (*n* = 3) by whole-body coronal maximum intensity projections (MIPs).

The capability of [^89^Zr]Zr-DFO-ToxAb to discern between infection and inflammation processes was compared to that of [^18^F]FDG, one of the most useful nuclear medicine tools in infectious-inflammatory pathology. [^18^F]FDG images 72 h after the inoculation of bacteria showed similar uptake in both the left and right leg muscles (Figure 4(a)), with a Log10-infected to Log10-inflamed ratio of 1.08 ± 0.01 (Figure 4(b)). This result is in accordance with the well-known limitations of FDG in distinguishing between infection and aseptic inflammatory processes. In contrast, at the same postinoculation time, [^89^Zr]Zr-DFO-ToxAb clearly and selectively accumulated in the infectious focus (Figure 4(a)), with a Log10-infected to Log10-inflamed ratio of 2.58 ± 0.38 (Figure 4(b)). These differences in the ratio between tracers support their ability to discern between both pathologies (Mann–Whitney *U* test, *p* < 0.05).

### 3.7. In Vivo PET/CT Imaging of Free [^89^Zr]Zr-Oxalate in Staphylococcal Infection-Inflammation Rat Model

As control of the free radionuclide uptake and biodistribution, we underwent PET/CT imaging at 24 and 48 h after the administration of the [^89^Zr]Zr-oxalic in the staphylococcal infection-inflammation model (*n* = 3). Nuclear imaging showed the main accumulation of the radionuclide in the bone as well as the irrelevant uptake in soft tissue (Figure 4 and Figure S4), especially compared to the [^89^Zr]Zr-DFO-ToxAb tracer.

### 3.8. In Vivo PET/CT Imaging of [^89^Zr]Zr-DFO-ToxAb in an Infective Osteoarthritis Mouse Model

**Figure 5:**
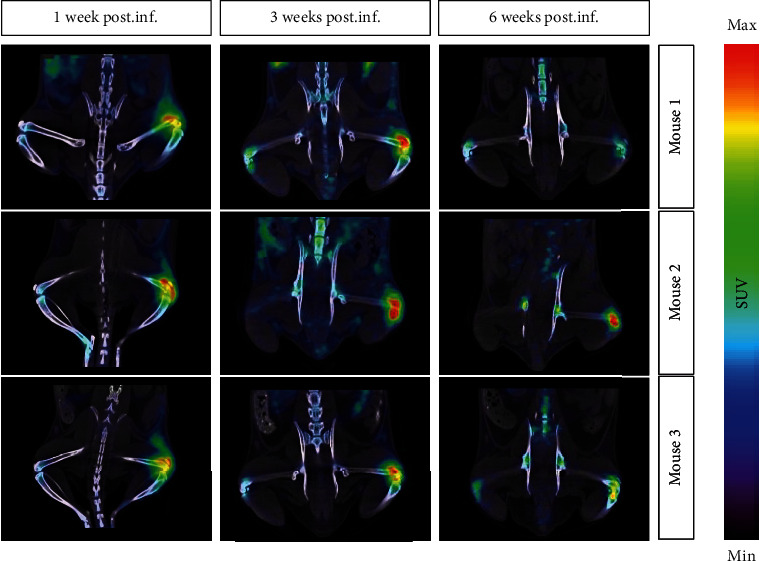
PET/CT in vivo imaging (*n* = 4) of [^89^Zr]Zr-DFO-ToxAb in infected osteoarthritis animal model 24 h (weeks 1 and 3 of infection) and 7 days (week 6 of infection) after tracer administration. In the images, the left joint corresponds to a healthy joint and the right joint corresponds to an infected joint.

PET imaging (*n* = 4) showed a high uptake of [^89^Zr]Zr-DFO-ToxAb in the joint area and in the surrounding soft tissues at week 1 of infection. At week 2 of infection, uptake of the radiotracer is still intense and more localized in the joint area (Figure 5, and Figure S6). In healthy joints, only a slight uptake is observed, corresponding to the uptake of free [^89^Zr], as observed in previous studies.

### 3.9. Validation of Infective Osteoarthritis Mouse Model

**Figure 6:**
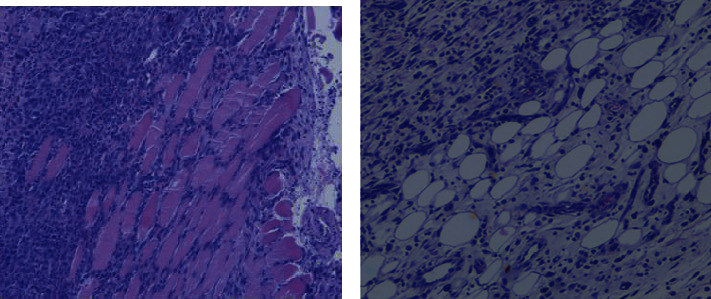
H&E stain of infective osteoarthritis tissue. (a) Muscle tissue. Infiltrated muscle can be observed on the right side of the image. (b) Soft tissue. Inflammation can be observed in soft tissue.

Histological analysis 6 weeks after surgery showed mild inflammation in the joint, bone, and muscle (Figure 6). On the other hand, microbiological analysis of the femur confirmed the presence of *S. aureus* in all femurs of mice with infected implants.

## 4. Discussion

Although promising advances are being made in the localization of staphylococcal infectious foci, selective and sensitive approaches for noninvasive detection are still lacking. One of the more clinically relevant issues is the inability to differentiate inflammatory from infectious processes, which may lead to false-positive diagnoses [19]. Motivated by the promising results obtained with immuno-PET imaging in the oncology field [38], the current study presents the evaluation of an immuno-PET tracer based on an ^89^Zr-radiolabeled anti-staphylococcal *α*-toxins antibody.

In vivo pharmacokinetic assessment of our tracer indicated a blood half-life of 2.3 days in rats, similar to that of other IgG-based radiotracers [39, 40]. Hepatic clearance and decreased activity values in the spleen and kidneys indicate hepatobiliary metabolism and elimination, confirming the usual behavior for full-length antibody immunotracers in rodents [26, 41]. Despite the metabolization of the [^89^Zr]Zr-DFO-ToxAb in these organs, its limited accumulation in nonspecific tissues supposes a surprising finding compared to biodistribution results previously reported for monoclonal antibody-based immuno-PET tracers [27].

In vivo evaluation of [^89^Zr]Zr-DFO-ToxAb as a diagnostic tool in PET/CT imaging of animal models with staphylococcal infection suggested the selectivity of our tracer. Assessment of the radioactive probe's ability to distinguish infection and inflammation by in vivo imaging offered promising results, as it showed significantly higher uptake in the infective region than with the inflammatory process provoked by the injection in the contralateral side at all observed time points. Demonstration of the presence of a noninfectious inflammatory focus has been achieved not only by histology (Figure 3) but also by in vivo FDG imaging. The FDG uptake suggested the presence of macrophages and inflammatory cells [42, 43]. The fact that the [^89^Zr]Zr-DFO-ToxAb tracer showed no uptake in this noninfectious inflammatory focus pointed out that the Fc reporter on the macrophages or other immune cells is not likely to be mediating the signal. In addition, specific accumulation of [^89^Zr]Zr-DFO-ToxAb against the free radionuclide in soft tissue was corroborated, showing the main uptake and biodistribution of [^89^Zr]Zr-oxalic in bone.

Regarding the selectivity of our tracer, although in vivo imaging at 1 h postadministration showed abundant circulation of the tracer in the bloodstream, its accumulation in the infected soft tissue is already significant, which compares favorably to the optimal imaging time established for other tracers designed for the detection of *S. aureus*-derived infections [23, 27]. We also observe a progressive accumulation of tracer in the infected soft tissue at the early stages of infection, which remains constant over time. These results are consistent with a purported mechanism of *α*-toxin secretion, which claims that greater amounts of this toxin may be secreted early to establish the infectious niche, and then diminish to avoid provoking a large immune system response [10]. Anyhow, the authors would like to remark on the limited number of works studying the biological behavior of this hemolysin. Although the selectivity of the radiotracer was assessed and validated in a staphylococcal infection-inflammation model, uptake may be altered by the possible irrigation of blood in the infected tissue caused by the pyogenic course of infection leading to abscess formation or the formation of a bacterial biofilm. To further confirm the capability of [^89^Zr]Zr-DFO-ToxAb to selectively detect active infections, we also performed the evaluation in a localized mouse model of infective osteoarthritis. In this model, the lack of irrigation may be due to the ischemic event happening to the osteomyelitis inside the bone. However, a high uptake of our radiotracer is observed in the infected joints, increasing in the second week of the study and decreasing over time. Therefore, we have proven the targeting of our radiotracer towards *S. aureus* in a model with no possible irrigation interference.

**Figure 7:**
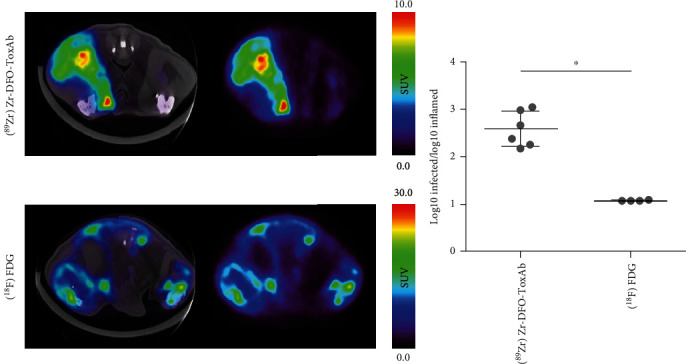
Comparison of [^89^Zr]Zr-DFO-ToxAb and [^18^F]FDG radiotracers in a local infection model. (a) PET/CT axial images from animals injected with [^89^Zr]Zr-DFO-ToxAb (*n* = 6; up) and [^18^F]FDG (*n* = 4; down), 72 h after the inoculation of bacteria. (b) Region of interest quantification, presented as the ratio of Log10-infected vs. Log10-inflamed ratio for the radiotracers [^89^Zr]Zr-DFO-ToxAb (*n* = 6) and [^18^F]FDG in infected and inflamed tissue (*n* = 4). Data are expressed as the mean ± standard deviation, and the threshold for significance was set at ^∗^*p* < 0.05.

Although other probes based on synthetic molecules have been developed for the in vivo detection of staphylococcal infection by molecular imaging [44], their translation to humans still needs to overcome some limitations, mainly those associated with false positives derived by their incapacity to discriminate sterile inflammation from infection and the tissue penetration limit for near-infrared light in the case of optical probes [45]. The ability of [^89^Zr]Zr-DFO-ToxAb to discern between both processes was not observed in the [^18^F]FDG studies (Figure 7), the present gold standard radiotracer for clinical diagnosis [46]. This observation highlights the potential clinical translation of the immunotracer, overcoming the main limitations of the current due to the nonspecific uptake of activated immune cells [47]. In addition, the development of a pathogen-specific probe can potentially guide physicians in the diagnostic process or therapeutic decision-making, providing highly valuable information. These results supported a possible use of [^89^Zr]Zr-DFO-ToxAb in monitoring and optimizing antibiotic treatment, enabling clinicians to establish treatment endpoints in patients requiring prolonged antibiotic use.

The transfer of our methodology to humans is further supported by the current application of the humanized version (the commercial MEDI4893) of the antibody used in our study in the treatment of staphylococcal pneumonia [48, 49], as well as by the extensive use of other ^89^Zr-based tracers in other fields, such as oncology [50]. Although this immunotherapy has shown some therapeutic limitations in phase III [51], the specificity and selectivity of the antibody towards *S. aureus* infections have been well demonstrated in numerous studies. In addition, this methodology based on labeling commercial antibodies simplifies the generation of selective bacterial tracers in contrast to other studies based on small molecules [20, 21], where the synthesis of the necessary precursors involves complex chemistry and may impair possibilities of clinical transfer.

Our study suffers from several limitations. First, from a microbiological point of view, in vitro studies employing different staphylococcal pathogens would be advisable to confirm the selectivity and specificity of [^89^Zr]Zr-DFO-ToxAb towards any *S. aureus* strain. However, the limitations derived from the nature of the target do not allow to perform these kinds of assessments. As the alpha-toxin is a target in suspension, rather than a receptor or fixed cell-bound biomolecule, the physical separation between the probe bound to the alpha-toxins in solution and the free probe is complex and difficult to achieve, which limits the reliability of results obtained with this type of assay. As a possible solution to assess these limitations, other assays such as surface plasma resonance or bead-based assays could be considered in further studies. In fields such as oncology, where the immuno-PET technique has been widely validated, anti-IgG antibody-based tracers have traditionally been used as control studies to confirm the selectivity of new immunotracers [52]. However, in the case of infectious processes, unlike in the oncological case, these IgG are abundantly present in the infectious site itself due to the immunological response. Therefore, this kind of approach cannot be used in the determination of immunotracer selectivity in infectious processes. These limitations have already been observed in other studies focused on the development of immunotracers for bacterial detection [27]. Second, an evaluation of the diagnostic capability of our tracer in other animal models that mimic more clinically relevant infectious diseases, such as infective endocarditis or pneumonia, is warranted to validate the diagnostic ability of the radiotracer in any staphylococcal infection. Finally, the long half-life of the radioisotope ^89^Zr, its high energies, and its production in cyclotrons could limit the application of the immunotracer. This latter limitation could be addressed by labeling the antibody with gallium-68. From a chemical point of view, we could use the same precursor (DFO-ToxAb) employed in the synthesis of [^89^Zr]Zr-DFO-ToxAb because the use of the molecule DFO for chelation of the short half-life isotope gallium-68 has been widely validated. It would not introduce modifications to the structure of the tracer; therefore, this approach should maintain the pharmacological properties of the tracer and its diagnostic capability, with the difference of using a radionuclide with a half-life of 68 min instead of 78 h. More importantly, from a biological standpoint, the high uptake of [^89^Zr]Zr-DFO-ToxAb observed at early time points (Figures 2(a) and 2(b)), even 1 h postinjection, clearly supports the use of this short half-life radionuclide in further experiments. This modification would significantly reduce not only the radiation received by the patient but also the possible application of the tracer in any hospital/biomedical facility due to the feasible production of the nuclide in small generators.

## 5. Conclusion

We have successfully synthesized an immuno-PET tracer capable of detecting *S. aureus* infections. This novel radiotracer is based on a radiolabeled antibody specific for staphylococcal alpha toxins, one of the main virulence factors associated with its lag-log and stationary phases. The in vivo assessment of our radiotracer, [^89^Zr]Zr-DFO-ToxAb, confirmed its ability to selectively detect staphylococcal infectious foci, allowing us to discern between infectious and inflammatory processes. The higher selectivity and specificity of the tracers compared to [^18^F]FDG supports the use of [^89^Zr]Zr-DFO-ToxAb for the noninvasive detection of infectious processes.

## Data Availability

The raw data and datasets generated and/or analyzed during the current study are available from the corresponding authors on reasonable request.

## Supplementary Materials

Supplementary Materials Figure S1: radiochemical purity of [^89^Zr]Zr-DFO-ToxAb assessed by radio-thin layer chromatography (radio-TLC). Table S1: ex vivo biodistribution results for infected versus inflamed soft tissue (*n* = 6). Figure S2: additional axial and coronal PET/CT images centered in the infected and inflamed tissues from the in vivo evaluation of [^89^Zr]Zr-DFO-ToxAb. Figure S3: immunohistochemistry of *S. aureus* in control and infected soft tissue. Upper row: hematoxylin and eosin staining. Lower row: histochemical staining. Blue: DAPI; green: antibody. Figure S4: additional coronal PET/CT images from the evaluation of [^89^Zr]Zr-oxalic biodistribution in local infection-inflammation model. Figure S5: experimental setup of in vivo PET/CT imaging of [^89^Zr]Zr-DFO-ToxAb in osteoarthritis mouse model. Figure S6: additional PET/CT in vivo images from the evaluation of [^89^Zr]Zr-DFO-ToxAb in infected osteoarthritis animal model at 1, 2, 3, 4, 5, and 6 weeks postinfection 24 h (weeks 1, 3, and 5 postinfection) or 7 days (weeks 2, 4, and 6 postinfection) after tracer administration.
