# Supplementary material for: In Vivo Detection of Staphylococcus aureus Infections Using Radiolabeled Antibodies Specific for Bacterial Toxins
Source: Int J Biomed Imaging. 2024 Apr 18;2024:3655327. doi: 10.1155/2024/3655327 (PMC11045290; doi:10.1155/2024/3655327)

*Research article*

**In vivo detection of *Staphylococcus aureus* infections using radiolabeled antibodies specific for bacterial toxins**

**María Isabel González ^1,2§^, Mario González-Arjona ^1§^, Lorena Cussó ^1,2,3,4^, Miguel Ángel Morcillo ^5^, John Jairo Aguilera-Correa ^6,7^, Jaime Esteban ^7^, Martha Kestler ^8^, Daniel Calle ^2^, Carlos Cerón ^9^, Marta Cortes-Canteli ^9,10^, Patricia Muñoz ^4,8,11^, Emilio Bouza ^4,8,11^, Manuel Desco ^1,2,3,4*^, Beatriz Salinas ^1,2,3,4*^**

* Correspondence: Beatriz Salinas (bsalinas@hggm.es) and Manuel Desco (desco@hggm.es); Instituto de Investigación Sanitaria Gregorio Marañón (IiSGM), Hospital General Universitario Gregorio Marañón de Madrid. Dr. Esquerdo, Spain 46. 28007 Madrid, Spain

^§^ María Isabel González and Mario González Arjona have contributed equally to this work.

Authors provide the following supplementary data to support the experimental results reported in the main article:

**· Figure S1:** Radiochemical purity of [^89^Zr]Zr-DFO-ToxAb assessed by radio-thin layer chromatography (radioTLC)


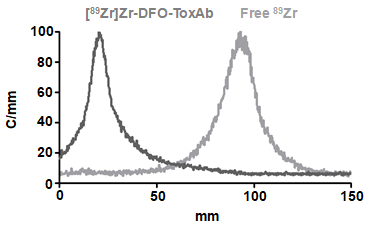


**· Table S1:** Ex vivo biodistribution results for infected versus inflamed soft-tissue (n = 6)

|  | Infected soft tissue  (% ID/g) | Inflamed soft tissue (% ID/g) | Ratio  infected vs. inflamed |
| --- | --- | --- | --- |
| 1 h post-injection | 0.61 ± 0.29 | 0.15 ± 0.03 | 4.02 |
| 24 h post-injection | 1.25 ± 0.83 | 0.14 ± 0.10 | 9.21 |
| 48 h post-injection | 0.74 ± 0.35 | 0.13 ± 0.10 | 5.80 |

**· Figure S2:** Additional axial and coronal PET/CT images centered in the infected and inflamed tissues from the in vivo evaluation of [^89^Zr]Zr-DFO-ToxAb


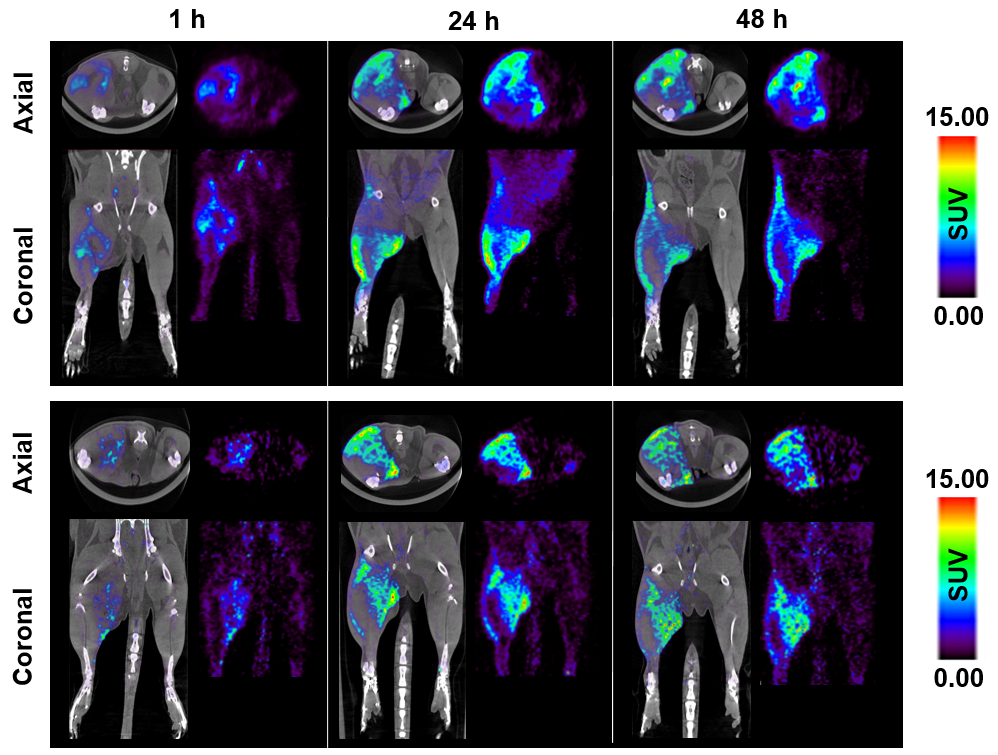


**· Figure S3:** Immunohistochemistry of *S. aureus* in control and infected soft-tissue. Upper row: Hematoxylin & Eosin staining. Lower row: Histochemical staining, Blue: Dapi, Green: Antibody


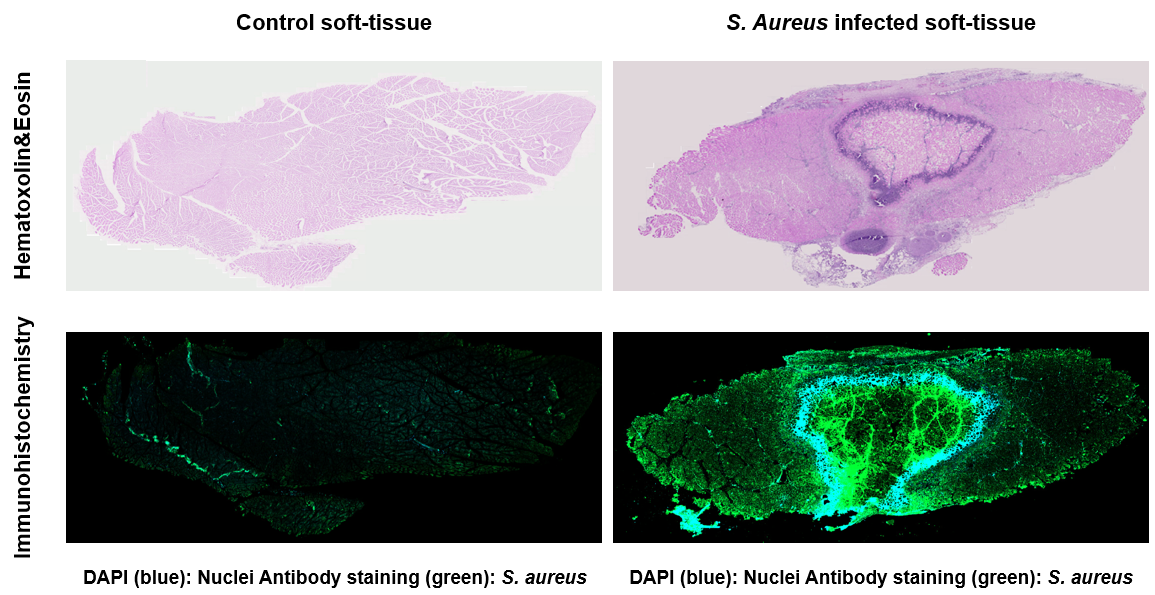


**· Figure S4:** Additional coronal PET/CT images from the evaluation of [^89^Zr]Zr-oxalic biodistribution in local infection-inflammation model


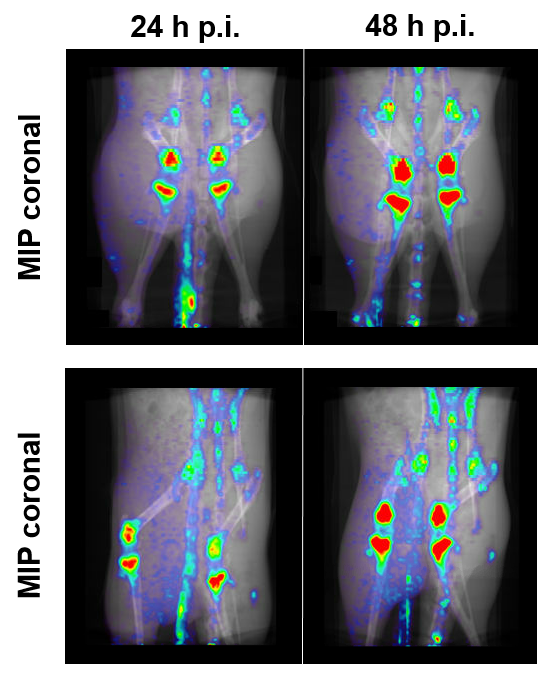


**· Figure S5:** Experimental setup of in vivo PET/CT imaging of [^89^Zr]Zr-DFO-ToxAb in osteoarthritis mice model.


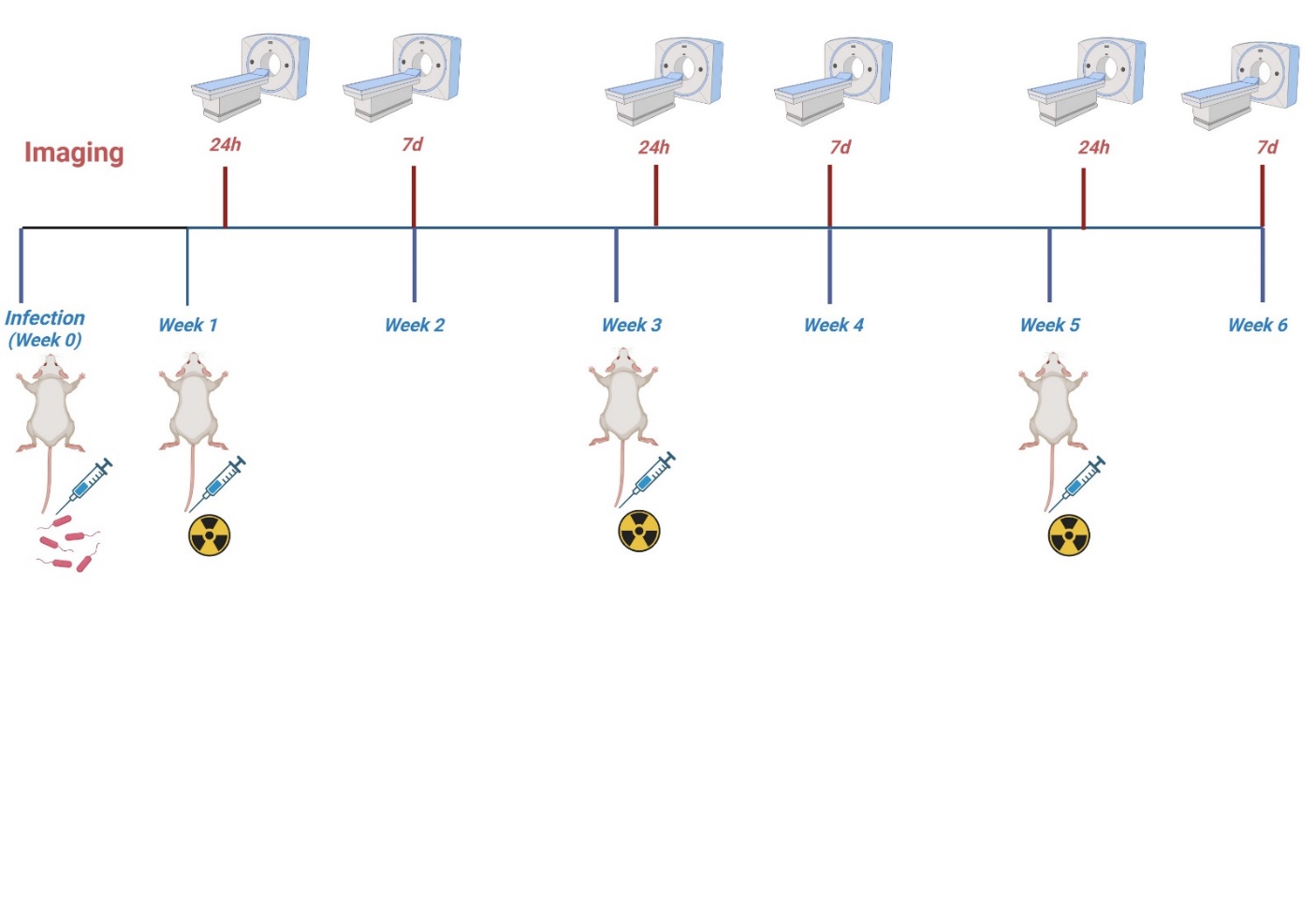


**· Figure S6:** Additional PET/CT in vivo images from the evaluation of [^89^Zr]Zr-DFO-ToxAb in infected osteoarthritis animal model, at 1, 2, 3, 4, 5 and 6 weeks post-infection 24h (weeks 1, 3 and 5 post-infection) or 7 days (weeks 2, 4 and 6 post-infection) after tracer administration.


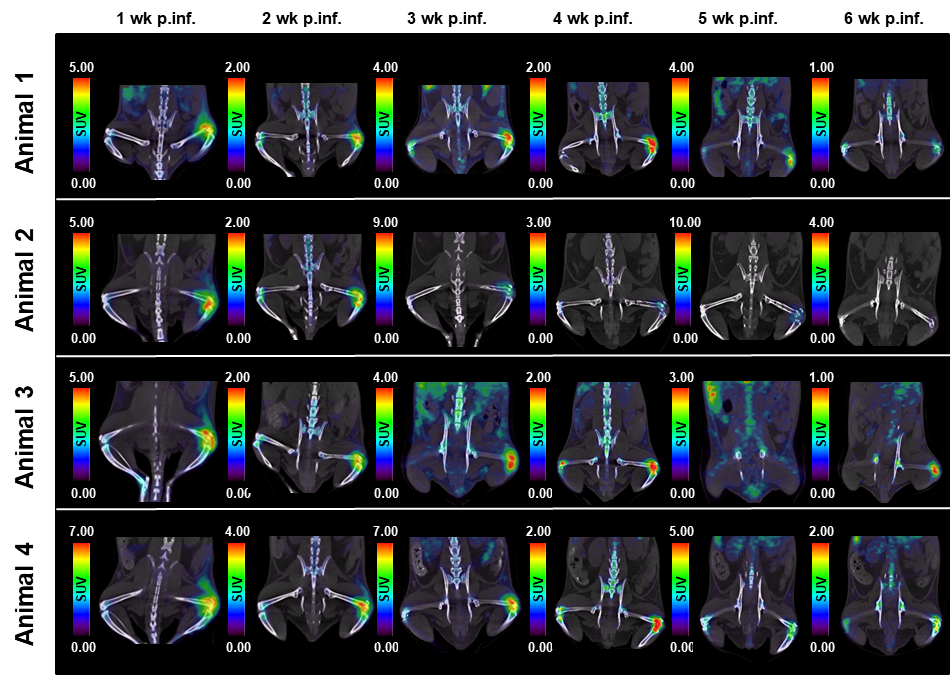

Supplement: Supplementary Materials — Figure S1: radiochemical purity of [89Zr]Zr-DFO-ToxAb assessed by radio-thin layer chromatography (radio-TLC). Table S1: ex vivo biodistribution results for infected versus inflamed soft tissue (n = 6). Figure S2: additional axial and coronal PET/CT images centered in the infected and inflamed tissues from the in vivo evaluation of [89Zr]Zr-DFO-ToxAb. Figure S3: immunohistochemistry of S. aureus in control and infected soft tissue. Upper row: hematoxylin and eosin staining. Lower row: histochemical staining. Blue: DAPI; green: antibody. Figure S4: additional coronal PET/CT images from the evaluation of [89Zr]Zr-oxalic biodistribution in local infection-inflammation model. Figure S5: experimental setup of in vivo PET/CT imaging of [89Zr]Zr-DFO-ToxAb in osteoarthritis mouse model. Figure S6: additional PET/CT in vivo images from the evaluation of [89Zr]Zr-DFO-ToxAb in infected osteoarthritis animal model at 1, 2, 3, 4, 5, and 6 weeks postinfection 24 h (weeks 1, 3, and 5 postinfection) or 7 days (weeks 2, 4, and 6 postinfection) after tracer administration. [file 3655327.f1.docx]
